# Supplementary material for: Hotspot DNA Methyltransferase 3A (DNMT3A) and Isocitrate Dehydrogenase 1 and 2 (IDH1/2) Mutations in Acute Myeloid Leukemia and Their Relevance as Targets for Immunotherapy
Source: Biomedicines. 2024 May 14;12(5):1086. doi: 10.3390/biomedicines12051086 (PMC11118067; doi:10.3390/biomedicines12051086)
Supplement: Supplementary file 1 [file biomedicines-12-01086-s001.zip › biomedicines-2938605-supplementary.pdf]

Supplementary Materials

Hotspot DNA Methyltransferase 3A (*DNMT3A*) and Isocitrate Dehydrogenase 1 and 2 (*IDH1/2*) Mutations in Acute Myeloid Leukemia and Their Relevance as Targets for Immunotherapy

Struckman *et al.*

Table S1. HLA class I typing of EBV-B cell lines.

EBV-B cell line	HLA-A		HLA-B		HLA-C	
HG00337	A*03:01	A*03:01	B*07:02	B*07:02	C*07:02	C*07:02
HG00366	A*02:01	A*24:02	B*07:02	B*15:01	C*03:03	C*07:02
HG00265	A*01:01	A*01:01	B*08:01	B*08:01	C*07:01	C*07:01
HG00103	A*02:01	A*11:01	B*35:01	B*40:01	C*03:04	C*04:01
JYW5098	A*02:01	A*02:01	B*07:02	B*07:02	C*07:02	C*07:02

HLA class I immunopeptidomics was performed on five EBV-B cell lines transduced with a minigene construct coding for *DNMT3A*, *IDH1* and *IDH2* hotspot mutations. The EBV-B cell lines were selected for expression of common HLA class I alleles present in at least 20% of European populations, i.e. HLA-A*01:01, A*02:01, A*03:01, A*24:02, B*07:02, B*08:01, C*07:01, C*07:02 and C*03:03/03:04.

Table S2. Selected peptides, transitions and collision energy measurements during parallel reaction monitoring mass spectrometry.

Precursor m/z	Fragment m/z	Sequence	Fragment ion	Peptide variant	Collision energy (%)
528,787838	245,185969	SPNGTIQNIL	y2	light	17
528,787838	698,34678	SPNGTIQNIL	b7	light	17
528,787838	812,389707	SPNGTIQNIL	b8	light	17
528,787838	925,473771	SPNGTIQNIL	b9	light	17
528,787838	406,698492	SPNGTIQNIL	b8	light	17
528,787838	463,240524	SPNGTIQNIL	b9	light	17
528,787838	449,243066	SPNGTIQNIL	a9	light	17
531,794742	245,185969	SPNGTIQNIL	y2	heavy	17
531,794742	704,360589	SPNGTIQNIL	b7	heavy	17
531,794742	818,403516	SPNGTIQNIL	b8	heavy	17
531,794742	931,48758	SPNGTIQNIL	b9	heavy	17
531,794742	409,705396	SPNGTIQNIL	b8	heavy	17
531,794742	466,247428	SPNGTIQNIL	b9	heavy	17
531,794742	452,249971	SPNGTIQNIL	a9	heavy	17
527,218931	890,367257	YTDVSNMSH	y8	light	26
527,218931	789,319579	YTDVSNMSH	y7	light	26
527,218931	674,292636	YTDVSNMSH	y6	light	26
527,218931	575,224222	YTDVSNMSH	y5	light	26
527,218931	265,118283	YTDVSNMSH	b2	light	26
527,218931	380,145226	YTDVSNMSH	b3	light	26
530,225835	896,381066	YTDVSNMSH	y8	heavy	26
530,225835	795,333388	YTDVSNMSH	y7	heavy	26
530,225835	680,306445	YTDVSNMSH	y6	heavy	26
530,225835	575,224222	YTDVSNMSH	y5	heavy	26
530,225835	265,118283	YTDVSNMSH	b2	heavy	26
530,225835	380,145226	YTDVSNMSH	b3	heavy	26
535,216388	906,362172	YTDVSNM[+15.994915]SH	y8	light	26
535,216388	805,314494	YTDVSNM[+15.994915]SH	y7	light	26
535,216388	690,287551	YTDVSNM[+15.994915]SH	y6	light	26
535,216388	591,219137	YTDVSNM[+15.994915]SH	y5	light	26
535,216388	265,118283	YTDVSNM[+15.994915]SH	b2	light	26
535,216388	380,145226	YTDVSNM[+15.994915]SH	b3	light	26
538,223293	912,375981	YTDVSNM[+15.994915]SH	y8	heavy	26
538,223293	811,328303	YTDVSNM[+15.994915]SH	y7	heavy	26
538,223293	696,30136	YTDVSNM[+15.994915]SH	y6	heavy	26
538,223293	591,219137	YTDVSNM[+15.994915]SH	y5	heavy	26
538,223293	265,118283	YTDVSNM[+15.994915]SH	b2	heavy	26
538,223293	380,145226	YTDVSNM[+15.994915]SH	b3	heavy	26
627,276977	1090,48335	YTDVSNM[+15.994915]SHLA	y10	light	26
627,276977	989,435671	YTDVSNM[+15.994915]SHLA	y9	light	26
627,276977	874,408728	YTDVSNM[+15.994915]SHLA	y8	light	26
627,276977	775,340314	YTDVSNM[+15.994915]SHLA	y7	light	26
627,276977	265,118283	YTDVSNM[+15.994915]SHLA	b2	light	26
627,276977	380,145226	YTDVSNM[+15.994915]SHLA	b3	light	26
630,785559	1097,500514	YTDVSNM[+15.994915]SHLA	y10	heavy	26
630,785559	996,452835	YTDVSNM[+15.994915]SHLA	y9	heavy	26
630,785559	881,425892	YTDVSNM[+15.994915]SHLA	y8	heavy	26
630,785559	782,357478	YTDVSNM[+15.994915]SHLA	y7	heavy	26
630,785559	265,118283	YTDVSNM[+15.994915]SHLA	b2	heavy	26
630,785559	380,145226	YTDVSNM[+15.994915]SHLA	b3	heavy	26
619,27952	1074,488435	YTDVSNMSHLA	y10	light	26
619,27952	973,440756	YTDVSNMSHLA	y9	light	26
619,27952	858,413813	YTDVSNMSHLA	y8	light	26
619,27952	759,345399	YTDVSNMSHLA	y7	light	26
619,27952	265,118283	YTDVSNMSHLA	b2	light	26
619,27952	380,145226	YTDVSNMSHLA	b3	light	26
622,788102	1081,505599	YTDVSNMSHLA	y10	heavy	26
622,788102	980,45792	YTDVSNMSHLA	y9	heavy	26
622,788102	865,430977	YTDVSNMSHLA	y8	heavy	26
622,788102	766,362563	YTDVSNMSHLA	y7	heavy	26
622,788102	265,118283	YTDVSNMSHLA	b2	heavy	26
622,788102	380,145226	YTDVSNMSHLA	b3	heavy	26

Figure S1. DNMT3A^{R882H} 9mer YTDVSNMSH is not presented on AML.

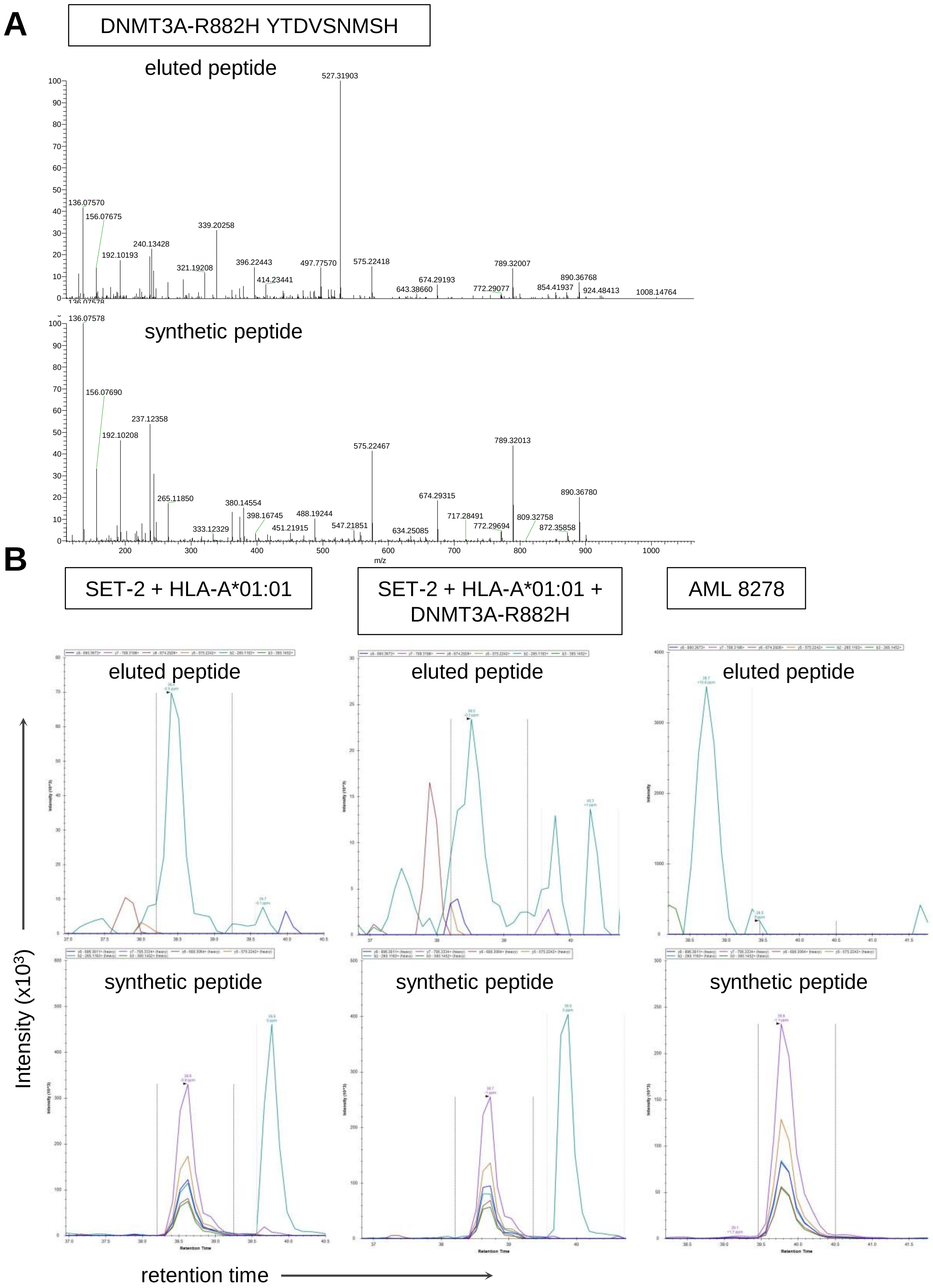

(A) The HLA-A*01:01-binding DNMT3A^{R882H}-derived 9-mer YTDVSNMSH peptide was eluted from EBV-B cell line HG00265 transduced with the retroviral minigene construct. Peptide sequence was identified by MS/MS and validated by comparing eluted and synthetic peptide mass spectra. **(B)** Mass chromatograms of YTDVSNMSH by PRM-MS. HLA eluates from AML cell line SET-2 (*DNMT3A^{R882H}*) transduced with HLA-A*01:01, SET-2 transduced with HLA-A*01:01 and the full-length *DNMT3A^{R882H}* gene and AML sample 8278 (*DNMT3A^{R882H}*, HLA-A*01:01) were injected into the mass spectrometer and spiked with 20 fmol YTD**V**SNMSH (heavy amino acid bold and underlined) as a reference.

Table S3. Predicted HLA class I-binding DNMT3A, IDH1, and IDH2 neopeptides.

Gene	Mutation	Prevalence	Predicted peptide	HLA	NetMHCpan 4.1 predicted binding		Wildtype variant	NetMHCpan 4.1 predicted binding (%Rank)	
DNMT3A	c.2645G>A / R882H	9.6%[16]	YTDVSNMS**H**LA	A*01:01	0.701	WB	YTDVSNMS**R**LA	1.089	WB
			YTDVSNMS**H**L	A*01:01	0.643	WB	YTDVSNMS**R**L	0.617	WB
			YTDVSNMS**H**	A*01:01	0.107	SB	YTDVSNMS**R**	0.438	SB
			VHYTDVSNMS**H**	A*01:01	1.389	WB	VHYTDVSNMS**R**	3.168	
			HYTDVSNMS**H**	A*01:01	0.808	WB	HYTDVSNMS**R**	1.885	WB
			MS**H**LARQRL	B*07:02	1.353	WB	MS**R**LARQRL	0.683	WB
			MS**H**LARQRL	B*08:01	1.569	WB	MS**R**LARQRL	1.164	WB
			S**H**LARQRLL	B*08:01	1.489	WB	S**R**LARQRLL	1.234	WB
			**H**LARQRLL	B*08:01	0.626	WB	**R**LARQRLL	2.895	
			YTDVSNMS**H**L	C*03:04	1.004	WB	YTDVSNMS**R**L	1.175	WB
			MS**H**LARQRL	C*03:04	0.381	SB	MS**R**LARQRL	1.525	WB
			S**H**LARQRLL	C*07:01	0.419	SB	S**R**LARQRLL	0.070	SB
			HYTDVSNMS**H**L	C*07:02	1.698	WB	HYTDVSNMS**R**L	2.423	
			S**H**LARQRLL	C*07:02	0.647	WB	S**R**LARQRLL	0.298	SB
	c.2644C>T / R882C	2.5%[16]	YTDVSNMS**C**LA	A*01:01	1.061	WB	YTDVSNMS**R**LA	1.089	WB
			YTDVSNMS**C**L	A*01:01	0.787	WB	YTDVSNMS**R**L	0.617	WB
			YTDVSNMS**C**	A*01:01	0.793	WB	YTDVSNMS**R**	0.438	SB
			S**C**LARQRLL	B*08:01	1.685	WB	S**R**LARQRLL	1.234	WB
			YTDVSNMS**C**L	C*03:04	1.323	WB	YTDVSNMS**R**L	1.175	WB
IDH1	c.394C>T / R132C	3.6%[17]	KPIIIG**C**HAY	B*07:02	1.390	WB	KPIIIG**R**HAY	0.762	WB
			KPIIIG**C**HA	B*07:02	1.526	WB	KPIIIG**R**HA	0.744	WB
	c.394C>G / R132G	0.6%[17]	IIG**G**HAYGDQY	A*01:01	1.807	WB	IIG**R**HAYGDQY	2.528	
			KPIIIG**G**HAY	B*07:02	1.023	WB	KPIIIG**R**HAY	0.762	WB
			KPIIIG**G**HA	B*07:02	0.947	WB	KPIIIG**R**HA	0.744	WB
	c.395G>A / R132H	0.4%[17]	IIG**H**HAYGDQY	A*01:01	1.997	WB	IIG**R**HAYGDQY	2.528	
			KPIIIG**H**HAY	B*07:02	1.054	WB	KPIIIG**R**HAY	0.762	WB
			KPIIIG**H**HA	B*07:02	0.669	WB	KPIIIG**R**HA	0.744	WB
			G**H**HAYGDQY	C*07:01	1.921	WB	G**R**HAYGDQY	0.302	SB
			G**H**HAYGDQY	C*07:02	1.082	WB	G**R**HAYGDQY	0.399	SB
	c.394C>A / R132S	0.4%[17]	IIIG**S**HAYGDQY	A*01:01	1.594	WB	IIIG**R**HAYGDQY	3.413	
			IIG**S**HAYGDQY	A*01:01	1.044	WB	IIG**R**HAYGDQY	2.528	
			IG**S**HAYGDQY	A*01:01	0.686	WB	IG**R**HAYGDQY	4.466	
			PIIIG**S**HAY	A*01:01	1.950	WB	PIIIG**R**HAY	3.330	
			G**S**HAYGDQY	A*01:01	0.345	SB	G**R**HAYGDQY	6.370	
			KPIIIG**S**HAY	B*07:02	1.053	WB	KPIIIG**R**HAY	0.762	WB
			KPIIIG**S**HA	B*07:02	0.652	WB	KPIIIG**R**HA	0.744	WB
IDH2	c.419G>A / R140Q	5.4%[18]	KSPNGTI**Q**NI	B*07:02	0.811	WB	KSPNGTI**R**NI	0.838	WB
			SPNGTI**Q**NIL	B*07:02	0.167	SB	SPNGTI**R**NIL	0.027	SB
			SPNGTI**Q**NI	B*07:02	0.192	SB	SPNGTI**R**NI	0.146	SB
	c.515G>A / R172K	2.1%[18]	ITIG**K**HAHGDQY	A*01:01	0.568	WB	ITIG**R**HAHGDQY	0.582	WB
			TIG**K**HAHGDQY	A*01:01	1.408	WB	TIG**R**HAHGDQY	1.461	WB
			VPGWTKPITIG**K**	A*03:01	0.983	WB	VPGWTKPITIG**R**	4.900	
			GWTKPITIG**K**	A*03:01	0.112	SB	GWTKPITIG**R**	1.139	WB
			WTKPITIG**K**	A*03:01	0.270	SB	WTKPITIG**R**	1.417	WB
			KPITIG**K**HAH	B*07:02	1.466	WB	KPITIG**R**HAH	1.108	WB
			KPITIG**K**HA	B*07:02	0.724	WB	KPITIG**R**HA	0.583	WB

Peptide sequences of 21 amino acids with the mutated amino acid encoded by *DNMT3A*, *IDH1* or *IDH2* hotspot mutations at position 11 were searched for 9-, 10- and 11-mer neopeptides with predicted binding to common HLA class I alleles with population frequencies above 20% in European populations by NetMHCpan4.1. Indicated is the mutation prevalence according to Ley et al. (2010) [16], Schnittger et al. (2010) [17] and Koszarska et al. (2013) [18]. Peptides with predicted binding ≤0.5%Rank are strong binders (SB), and peptides with predicted binding 0.5%>%Rank≤2.0% are weak binders (WB). Predicted HLA class I binding is also shown for wildtype peptide variants.

Figure S2. *DNMT3A* and *IDH2* mutations in SET-2 and K562-R140Q.

A. SET-2

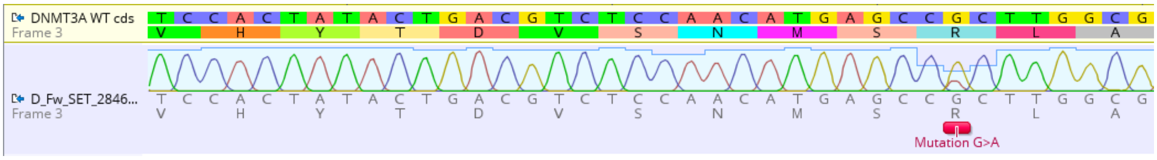

B. K562-R140Q

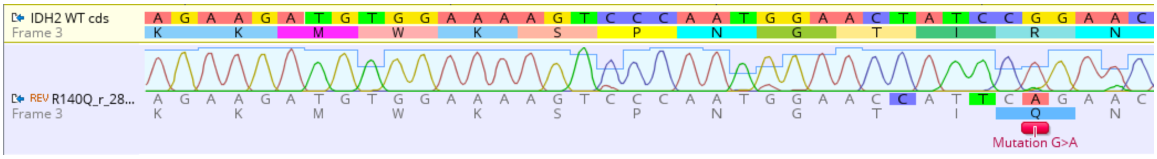

RNA was isolated and cDNA generated from AML cell line SET-2 (*DNMT3A*^{R882H}) and chronic myelogenous leukemia cell line K562-R140Q in which bi-allelic *IDH2*^{R140Q} mutations were introduced by CRISPR/Cas9 mediated genome editing [24]. Mutations were validated by targeted PCR and Sanger sequencing. **(A)** DNA sequence of the *DNMT3A* gene in SET-2 shows a heterozygous c.2645G>A (R882H) mutation. **(B)** DNA sequence of the *IDH2* gene in K562-R140Q shows a homozygous c.419G>A (R140Q) mutation.

Figure S3. Surface presentation of YTDVSNMSHLA and SPNGTIQNIL is not detected by PRM-MS on AML cells with *DNMT3A*^{R882H} or *IDH2*^{R140Q} mutations.

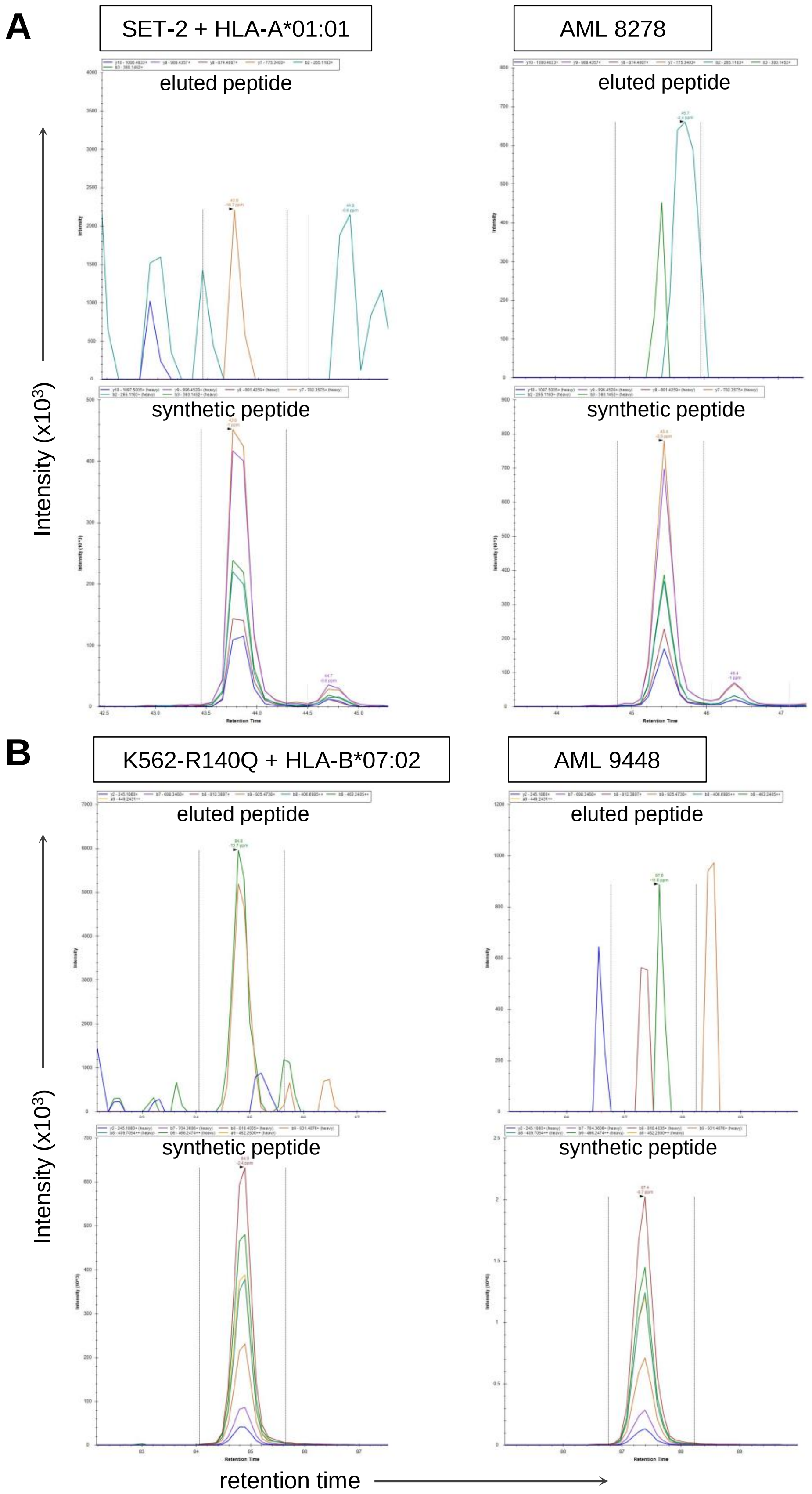

(A) Mass chromatograms showed no YTDVSNMSHLA in HLA-eluates of SET-2 (*DNMT3A*^{R882H}) transduced with HLA-A*01:01 and AML sample 8278 (*DNMT3A*^{R882H}, HLA-A*01:01). Heavy-isotope labeled YTDVSNMSH**L**A (heavy amino acid bold and underlined) was spiked-in as a reference. **(B)** Mass chromatograms showed no SPNGTIQNIL in HLA-eluates of K562-R140Q transduced with HLA-B*07:02 and AML sample 9448 (*IDH2*^{R140Q}, HLA-B*07:02). Heavy-isotope labeled S**P**NGTIQNIL was spiked-in as a reference.

Figure S4. HLA class I surface expression on AML samples and cell lines.

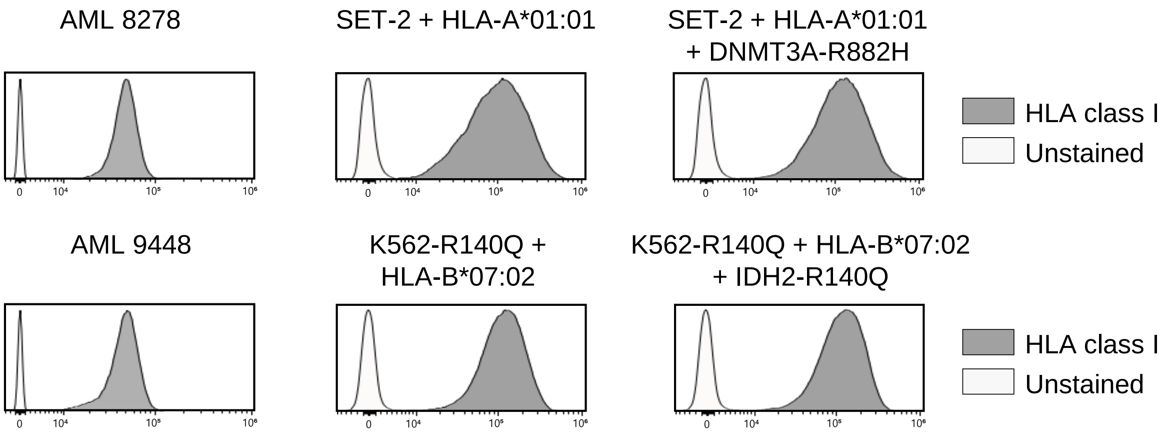

Mean fluorescence intensity after staining of patient AML samples and AML cell lines with a fluorescent antibody against HLA class I by flow cytometry.

Table S4. Isolation of DNMT3A^{R882H} -specific T-cell clones.

Donor	HLA-A		HLA-B		Reactivity against DNMT3A^{R882H} peptide	Reactivity against transduced DNMT3A^{R882H} gene
3	*01:01	*02:01	*07:02	*08:01	1	0
5	*01:01	*03:XX	*07:02	*08:01	1	0
8	*01:XX	*03:XX	*13:XX	*14:XX	3	1
10	*01:01	*03:01	*08:01	*35:01	0	
21	*01:01	*11:XX	*08:01	*56:01	0	
22	*01:01	*03:01	*08:01	*35:01	1	0
23	*01:01	*03:01	*08:01	*44:02	0	
24	*01:01	*03:01	*08:01	*13:02	0	
25	*01:01		*08:01		0	
30	*01:01	*03:01	*07:02	*08:01	0	
31	*01:01	*03:01	*35:01	*51:01	1	0
35	*01:01	*03:01	*07:02	*08:01	2	0
12	*01:XX	*03:XX	*07:XX	*18:XX	0	
16	*01:XX	*02:XX	*38:XX	*57:XX	0	
17-I^{C}	*01:XX	*24:XX	*07:02	*15:17	7	2
17-II^{C}	*01:XX	*24:XX	*07:02	*15:17	17	2
11	-	-	-	-	0	
13	-	-	-	-	0	
18	-	-	-	-	0	
19	-	-	-	-	0	
14	*11:01	*24:02	*35:01	*45:01	0	
15	*02:XX	*32:XX	*14:XX	*40:XX	0	
20	*03:XX	*24:XX	*14:01	*15:01	0	
32	*03:01	*25:01	*07:02	*18:01	0	
33	*02:01	*03:01	*07:02	*51:01	0	
34	*11:01	*68:01	*27:05	*57:01	0	
**26**					**43**	**5**

PBMC from 26 healthy individuals were screened for DNMT3A^{R882H} -specific CD8 T cells. Single cell sorted pHLA-tetramer-positive CD8 T-cell clones were isolated and screened for specific recognition of HLA-A*01:01 positive EBV-B cells loaded with YTDVSNMSHLA by IFN-γ ELISA. T-cell clones that were peptide-specific were tested for reactivity against K562 transduced with HLA-A*01:01 and the *DNMT3A*^{R882H} gene and HL-60 (HLA-A*01:01 positive) transduced with the *DNMT3A*^{R882H} gene. Five T cell clones from two healthy donors (donors 8 & 17) were reactive against the *DNMT3A*^{R882H} but not wildtype *DNMT3A* gene. Donor 17 has been screened twice (17-I^{C} & 17-II^{C}).

Table S5. Isolation of $IDH2^{R140Q}$-specific T-cell clones.

Donor	HLA-A		HLA-B		Reactivity against $IDH2^{R140Q}$ peptide	Reactivity against the transduced $IDH2^{R140Q}$ gene
1	*03:01	*26:01	*07:02	*27:05	1	1
2	*02:01	*03:01	*07:02	*51:01	0	
3	*01:01	*02:01	*07:02	*08:01	0	
4	*02:01	*03:01	*07:02	*44:02	0	
5	*01:01	*03:XX	*07:02	*08:01	1	1
6	*02:01	*03:01	*07:02	*51:01	1	0
7	*02:01	*03:01	*07:02	-	1	0
17-I[C]	*01:XX	*24:XX	*07:02	*15:17	0	
17-II[C]	*01:XX	*24:XX	*07:02	*15:17	0	
28	*03:01	*11:01	*07:02	*40:02	0	
30	*01:01	*03:01	*07:02	*08:01	1	0
32	*03:01	*25:01	*07:02	*18:01	0	
33	*02:01	*03:01	*07:02	*51:01	1	1
35	*01:01	*03:01	*07:02	*08:01	0	
12	*01:XX	*03:XX	*07:XX	*18:XX	0	
26	*02:XX	*11:XX	*07:XX	*15:XX	0	
11	-	-	-	-	0	
13	-	-	-	-	0	
18	-	-	-	-	0	
19	-	-	-	-	0	
9	*01:XX	*11:XX	*08:XX	*13:XX	0	
10	*01:01	*03:01	*08:01	*35:01	0	
14	*11:01	*24:02	*35:01	*45:01	0	
15	*02:XX	*32:XX	*14:XX	*40:XX	0	
16	*01:XX	*02:XX	*38:XX	*57:XX	0	
20	*03:XX	*24:XX	*14:01	*15:01	0	
27	*03:XX	*68/69:XX	*15:01	*27:XX	0	
29	*02:01	*03:01	*35:01	*57:01	0	
31	*01:01	*03:01	*35:01	*51:01	0	
34	*11:01	*68:01	*27:05	*57:01	0	
**30**					**5**	**3**

PBMC from 30 healthy individuals were screened for $IDH2^{R140Q}$-specific CD8 T cells. Single cell sorted pHLA-tetramer-positive CD8 T-cell clones were isolated and screened for specific recognition of HLA-B*07:02 positive EBV-B cells loaded with SPNGTIQNIL by IFN-γ ELISA. T-cell clones that were peptide-specific were tested for reactivity against K562 transduced with HLA-B*07:02 and the full-length $\mathit{IDH2}^{R140Q}$ gene. Three T-cell clones were reactive against the $\mathit{IDH2}^{R140Q}$ but not wildtype *IDH2* gene.

Figure S5. Gating strategy for isolation of DNMT3A^{R882H} or IDH2^{R140Q}-specific CD8 T cells by flow cytometry.

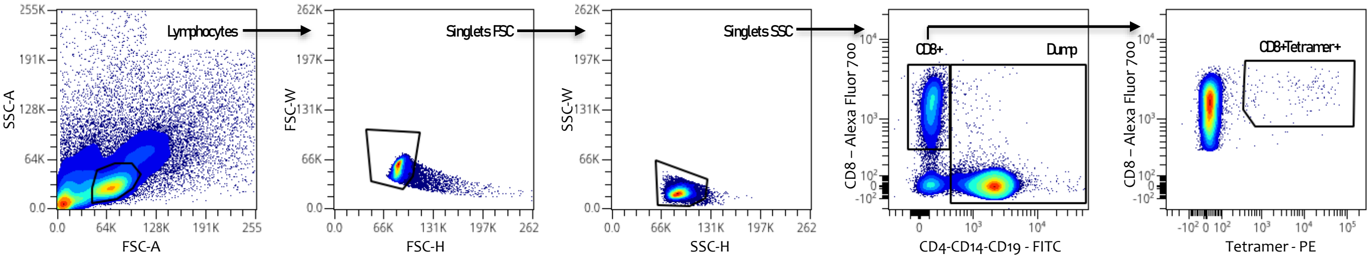

PBMC enriched for pHLA-tetramer-positive CD8 T cells were gated for single-cell lymphocytes that were negative for CD4, CD14 and CD19 (dump). pHLA-tetramer positive T cells within the CD8 gate were single-cell sorted by FACS.

Figure S6. Recognition of patient-derived AML samples by T-cell clone 8.6F10.

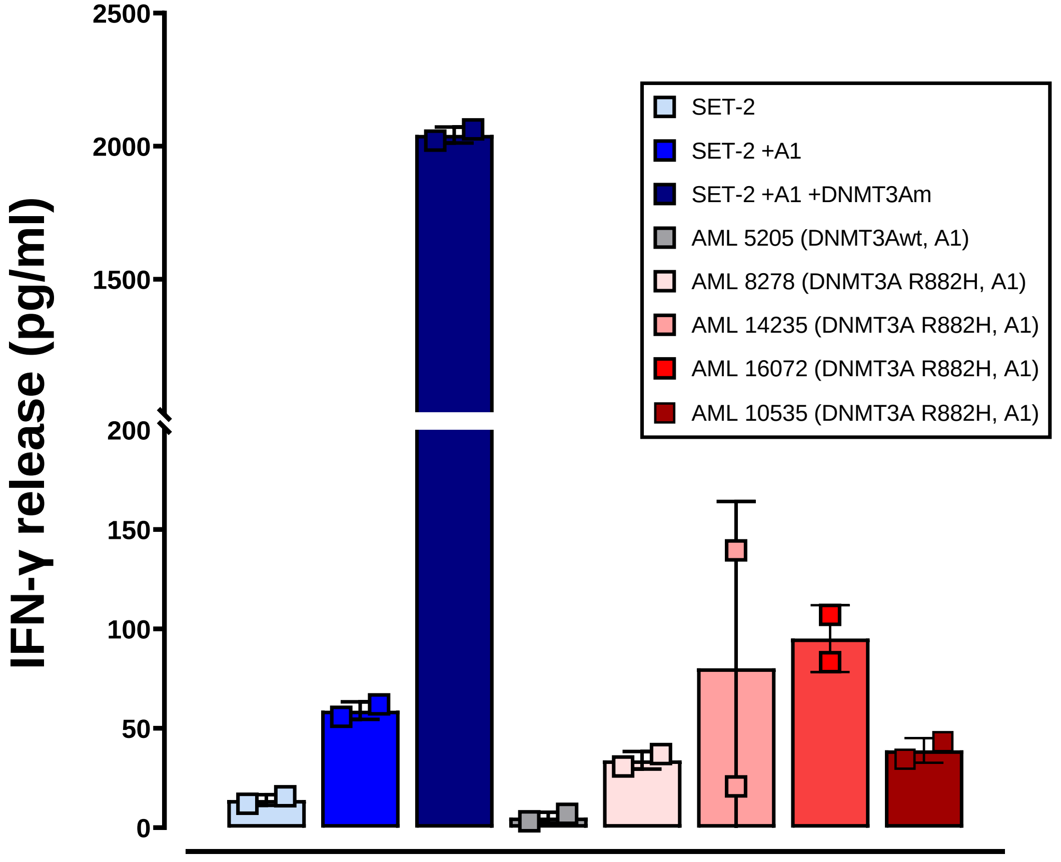

Reactivity of T-cell clone 8.6F10 was tested against AML cell line SET-2 (*DNMT3A*^{R882H}), SET-2 transduced with HLA-A*01:01 (SET-2 +A1), SET-2 transduced with HLA-A*01:01 and the full-length *DNMT3A*^{R882H} gene (SET-2 +A1 +DNMT3Am) and four HLA-A*01:01 and *DNMT3A*^{R882H} patient-derived AML samples 8278, 14235, 16072 and 10535. Patient-derived AML 5205 (HLA-A*01:01, wildtype *DNMT3A*) was included as negative control. IFN-γ release in duplicate wells of a single experiment are shown.

Figure S7. Surface expression of HLA class I and adhesion molecules on AML patient samples.

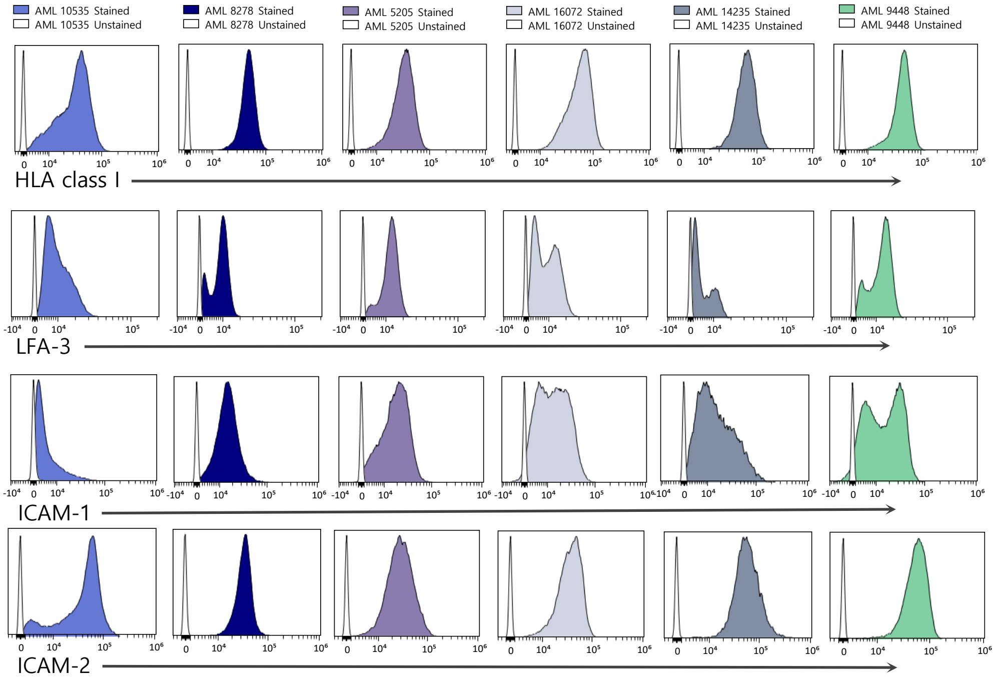

Mean Fluorescence Intensity (MFI) of patient AML samples stained with fluorescent antibodies against HLA class I, LFA-3 (Lymphocyte Function-Associated antigen 3), ICAM-1 (InterCellular Adhesion Molecule-1) or ICAM-2 (InterCellular Adhesion Molecule-2) by flow cytometry. Unstained samples are shown as controls.
